# Conducting Precision Medicine Research with African Americans

**DOI:** 10.1371/journal.pone.0154850

**Published:** 2016-07-21

**Authors:** Chanita Hughes Halbert, Jasmine McDonald, Susan Vadaparampil, LaShanta Rice, Melanie Jefferson

**Affiliations:** 1 Department of Psychiatry and Behavioral Sciences, Medical University of South Carolina, Charleston, SC, United States of America; 2 Hollings Cancer Center, Medical University of South Carolina, Charleston, SC, United States of America; 3 Ralph H. Johnson Veteran’s Administration Medical Center, Charleston, SC, United States of America; 4 Department of Epidemiology, Columbia University, Mailman School of Public Health, New York, NY, United States of America; 5 Moffitt Cancer Center, Tampa, FL, United States of America; Duke University, UNITED STATES

## Abstract

**Importance:**

Precision medicine is an approach to detecting, treating, and managing disease that is based on individual variation in genetic, environmental, and lifestyle factors. Precision medicine is expected to reduce health disparities, but this will be possible only if studies have adequate representation of racial minorities.

**Objective:**

It is critical to anticipate the rates at which individuals from diverse populations are likely to participate in precision medicine studies as research initiatives are being developed. We evaluated the likelihood of participating in a clinical study for precision medicine.

**Design, Setting, Participants:**

Observational study conducted between October 2010 and February 2011 in a national sample of African Americans.

**Main Outcome Measure:**

Intentions to participate in a government sponsored study that involves providing a biospecimen and generates data that could be shared with other researchers to conduct future studies.

**Results:**

One third of respondents would participate in a clinical study for precision medicine. Only gender had a significant independent association with participation intentions. Men had a 1.86 (95% CI = 1.11, 3.12, p = 0.02) increased likelihood of participating in a precision medicine study compared to women in the model that included overall barriers and facilitators. In the model with specific participation barriers, distrust was associated with a reduced likelihood of participating in the research described in the vignette (OR = 0.57, 95% CI = 0.34, 0.96, p = 0.04).

**Conclusion and Relevance:**

African Americans may have low enrollment in PMI research. As PMI research is implemented, extensive efforts will be needed to ensure adequate representation. Additional research is needed to identify optimal ways of ethically describing precision medicine studies to ensure sufficient recruitment of racial minorities.

## Introduction

On January 20, 2015, a new precision medicine initiative (PMI) was announced by President Barack Obama [1]. The long-term goal of the PMI is to develop and implement individualized approaches to disease prevention and treatment based on genomic and molecular information as well as environmental and lifestyle factors. To realize this goal, new research will be needed to develop genomic approaches to medical care and to evaluate the effects of these strategies on health care outcomes. A research cohort has been proposed to achieve the goals of PMI; this cohort is likely to be based on participants previously enrolled in cohorts funded by the NIH [1]. While this approach represents an efficient strategy for recruiting participants for new PMI research, the racial and ethnic diversity of these cohorts will need to be examined carefully to ensure that the individualized approaches developed through the PMI do not exacerbate disparities in health outcomes. This is because racial and ethnic minorities are likely to be under-represented in existing cohorts and biobanks established to support genomic research. Using data from published genome wide association studies (GWAS), Haga found that 92% of participants in GWAS research conducted in the United States were white and only 3% were African American [2].

Research is now being conducted to identify and understand barriers and facilitators to African American participation in genomics research; most recently, we examined these issues within the context of cancer genetics research [3,4]. Our research demonstrates that African Americans would consider the potential benefits to themselves, relatives, and community as part of making a decision to participate in cancer genetics research, but concerns about exploitation, distrust of researchers, and investigators' motives are also important to participation decisions [3]. African Americans would also consider who has access to their personal information and what would happen to these data when making a decision to participate in cancer genetics research [3]. Despite these concerns, however, 73% of African Americans would participate in a cancer genetics research study [5] and 23% would be very likely to donate a biospecimens to a biobank [4]. But, the expectations that individuals have about what would happen to their biospecimen were important to biobank donation intentions. Specifically, respondents who had greater positive expectations about participating in cancer genetics research and reported more participation facilitators relative to barriers were most likely to be willing to donate to a biobank [4].

The expectations that individuals have about research are shaped by many factors that include what potential participants are told about the procedures involved in participation and how the study is described as part of obtaining informed consent. While prior studies have examined barriers and facilitators to minority participation in medical research [6], the PMI is a new initiative that is anticipated to have several unique features that include the creation of a national cohort that includes research subjects who have volunteered to provide genetic data, donate biological samples, and contribute information about their lifestyle that is linked with their electronic health record. This new model of conducting research raises several issues related to the engagement of research volunteers, data sharing, and privacy; because of the under-representation of racial minorities in genomic research [2], attention is now being given to ensuring the racial and ethnic diversity of the PMI cohort [7]. For instance, if PMI cohorts are established from existing studies, then the distribution of racial and ethnic minorities is likely to reflect the diversity of established cohorts. Further, individuals being recruited to participate in the PMI from established cohorts may have more positive expectations and be more likely to participate in a new study, depending on their experiences in the original cohort. But, African Americans are likely to be under-represented in existing cohorts [2]; therefore, they may not be readily able to base expectations about participating in PMI studies on prior experiences. For this reason, their decisions about participating in PMI research may be based on how the study is described and the procedures involved in participation. To enhance the efficiency of recruiting African Americans ethically into PMI studies, it is important to evaluate their willingness to participate in genomic research based on how projects might be described. Therefore, in this study, we examined the likelihood that African Americans would participate in a clinical study that was methodologically similar to the cohort research that has been proposed as part of PMI [1]. We were also interested in identifying variables that had a significant independent association with participating in the clinical study that was described in the vignette.

## Materials and Methods

This study was reviewed and approved by the Institutional Review Board (IRB) at the University of Pennsylvania and was also reviewed and approved by the IRB at the Medical University of South Carolina after the Principal Investigator (CHH) moved to this institution. Verbal informed consent was obtained from all participants. Participants completed a telephone-based survey, thus written consent was not obtained. Participant consent was recorded using a screening questionnaire. We developed a research vignette that was based on findings from our qualitative research on decision making about participating in cancer genetic research [3]. Specifically, the vignette asked respondents how likely it was that they would participate in a study (1 = unlikely to 5 = very likely) that: (1) was sponsored by the government to provide information about the health of African Americans, (2) participation involves answering a questionnaire and providing a cheek swab, (3) the data will be used for the current study and also may be shared with other researchers to conduct future studies, (4) participants would not receive any results. This was one of eight vignettes administered to African American adults enrolled into a national survey study of attitudes about participating in cancer genetics research. Survey details have been described previously [4] and this report includes 510 survey respondents (34% of the total sample) who provided data on their willingness to participate in this research vignette.

### Measures

Our analysis was guided by findings from prior studies on African American participation in genetics research [8–10] and our previous research on biobanking among African Americans which has shown that distrust in health care providers, attitudes about genetics research, and perceived barriers and facilitators to participating in research are associated with intentions to donate biospecimens to a biobank [4]. Specifically, we measured attitudes about participating in genetics research using items that asked respondents how much they agreed or disagreed (1 = strongly disagree, 2 = disagree, 3 = neutral, 4 = agree, 5 = strongly agree) with statements that assessed positive outcomes (e.g., the results would be used to help future generations, I would get information about how to detect, prevent, and treat cancer that was just for me) and negative outcomes related to privacy (e.g., my information would not be kept private), and impact on health care and outcomes (e.g., the results would be used to develop cancer treatment drugs that someone like me could not afford). We summed responses to these items so that higher scores reflected greater expectations about positive and negative outcomes. The Cronbach’s alpha for these scales were 0.5 for positive expectations, 0.65 for negative expectations about privacy, and 0.55 for negative expectations about the impact on health care and outcomes in our previous research. To measure participation barriers and facilitators, respondents were asked how much issues such as concerns about disclosure of personal information (e.g., blood or saliva sample, medical history) and provision of free medication or health care would affect their decision to participate in cancer genetics research (1 = very likely, 2 = somewhat likely, 3 = make no difference, 4 = somewhat unlikely, 5 = very unlikely). Responses of ‘somewhat or very unlikely’ were recoded as -1, responses of ‘make no difference’ were recoded as 0, and responses of ‘somewhat or very likely’ were recoded as 1. We summed responses to the recoded items to calculate a total score such that a negative score indicated greater barriers, a positive score indicated greater facilitators, and a score of zero indicated an equal number of barriers and facilitators. Forty-five percent of respondents reported more participation facilitators, 9% of respondents had an equal number of barriers and facilitators, and 46% of respondents reported more participation barriers. Lastly, we used the altruism scale to measure the extent to which respondents were concerned about the needs and benefits to others relative to their own [11]. Altruism items were as follows: (1) people should be willing to help others who are less fortunate, (2) those in need have to learn to take care of themselves and not depend on others, (3) personally assisting people in trouble is very important to me, (4) these days people need to look after themselves and not overly worry about others. Items were summed so that higher scores reflected greater altruism; the Cronbach’s alpha for the scale was 0.40.

### Statistical Methods

First, descriptive statistics were generated to characterize respondents in terms of sociodemographic factors, attitudes about participating in genetics research, and participation barriers and facilitators. Frequencies were also generated to characterize willingness to participate in the study described in the research vignette. Next, we generated a multivariate logistic regression model to examine the independent associations between attitudes, barriers and facilitators, and participation intentions. Since individuals may be unwilling to donate biospecimens and personal health information to a biobank because of perceived barriers [4], we examined the association between reasons that would make respondents most unlikely to participate and intentions in a separate multivariate logistic regression model. Study data were weighted in these analyses to account for phone service (landline or cellular) and population targets for education, age, and gender using estimates from the March 2011 Current Population Survey.

## Results

S1 Table shows the characteristics of the study sample. Overall, 31% of respondents reported that it was very or somewhat likely that they would participate in a government-sponsored study that involved answering a questionnaire and providing a cheek swab, the data would be used for the current study and could also be shared with other researchers, and participants would not receive any results. Sixty-nine percent of respondents reported that it was very/somewhat unlikely or neutral that they would participate. As shown in S2 Table, there was high endorsement of items that measured positive expectations about participating in cancer genetics research and lower endorsement of those that measured negative expectations. For instance, 86% of respondents agreed that the results of would be used to help future generations and 84% agreed that they would feel that they contributed to the development of better strategies for detecting, preventing, and treating cancer. With respect to endorsement of items that measured negative expectations about privacy, only 34% agreed that researchers would use the results of the study to make profits and 16% agreed that their DNA would be tampered with. The endorsement of items that measured negative expectations about the impact of participating in cancer genetics research was similar: 57% agreed that the results of the study would be used to develop cancer treatment drugs that someone like them could not afford and 17% agreed that researchers would be dishonest about the purpose of the study. S3 Table shows descriptive information on reasons for participating in cancer genetics research. The most important participation barriers, or reasons for not participating in studies, included: if respondents did not know who would be able to obtain their personal information (60% unlikely to participate), if it were hard to get to where the study was being conducted (63% unlikely to participate), and if the findings from the study would not be made available (59% unlikely to participate).

S4 Table reports the results of the multivariate logistic regression analyses. Only gender had a significant independent association with the likelihood of participating in a PMI study in the model that included overall participation barriers and facilitators. Compared to women, men had 1.86 increased likelihood of participating in the PMI study (95% CI = 1.11, 3.12, p = 0.02). No other sociodemographic factors had a significant association with the likelihood of participation. While respondents who were distrustful of researchers had a lower likelihood of participation compared to those who trusted investigators, this association was not statistically significant (OR = 0.61, 95% CI = 0.36, 1.03, p = 0.06). Beliefs about positive expectations, concerns about privacy, and negative expectations about the impact of genetic research did not have significant associations with the likelihood of participation. The results of the logistic regression model that included specific participation barriers were similar. Male gender had a significant association with an increased likelihood of participation (OR = 1.85, 95% CI = 1.10, 3.10, p = 0.02) whereas distrust was associated with a reduced likelihood of participating in the research described in the vignette (OR = 0.57, 95% CI = 0.34, 0.96, p = 0.04).

## Discussion

As PMI research and cohorts are being developed, it is critical to anticipate the rates at which individuals from diverse populations are likely to participate. Racial and ethnic minorities are under-represented in genomics research [2]; their continued lack of participation in PMI studies will further exacerbate disparities in health care outcomes. Limited inclusion of racial and ethnic minorities is likely to result in sample sizes that are too small to support the discovery of genomic risk factors for disease and development of personalized therapeutic and prevention strategies involving and relevant to these underrepresented groups. Data on participation intentions allows investigators to anticipate the rates and which potential participants will provide informed consent for study enrollment. This data can also be used to project recruitment costs. Our study is the first to examine the likelihood that African American adults would participate in a government sponsored study that was methodologically similar to the cohort study that has been described as an example of PMI research. Other studies have evaluated barriers and facilitators to minority participation in medical research overall [6] and intentions to participate in genetic research [4,5]. But, research on intentions to participate in genetic research did not describe the procedures involved in participation, how study data would be used, nor indicate if results would be made available. This innovative aspect of our present study extends previous research on participation intentions in an important way by providing more contextual information about study procedures and the outcomes of enrolling in the research. However, our research is not without some limitations, including the cross-sectional collection of data on attitudes about participating in research, perceived barriers and facilitators to participation, and participation intentions and the landline and cellular response rates of 31.8% and 26.8%, respectively. Even with diminishing response rates, however, the samples enrolled in telephone surveys are likely to be similar to the US population in terms of sociodemographic characteristics [12]. Additional limitations may be that we measured participation intentions rather than actual enrollment rates in a subset of survey respondents. This was because specific PMI studies have not been developed and behavioral intentions are the best predictor of actual behavior. Our previous research on genetic testing decisions among African Americans suggests that intentions are a reasonable proxy for decision outcomes in this population [13,14]. The terminology we used to describe the study in the research vignette may be an additional limitation that may have generated a negative perception of the study described in the scenario. Thus, as PMI studies are implemented, it will be important to examine actual participation rates using longitudinal designs in which attitudes, barriers, and facilitators are measured prospectively before enrollment decisions are made based on the terminology used to describe studies as part of obtaining informed consent.

In contrast to prior studies that evaluated participation intentions in cancer genetics research and biobanks [4,5], and research showing that 73% of African Americans actually provided consent to have biologic samples and DNA stored in the NHANES repository for genetic research [15,16]; only about one-third of respondents in the present study would participate in a study that is methodologically similar to the cohort proposed as part of PMI research [1]. Our vignette described a potential benefit to African Americans, but individuals may still have privacy concerns that reduce participation in PMI research. In the present study, about one-third of respondents endorsed concerns that researchers would share personal information with others, information would not be kept private, and other individuals would be able to access their DNA. The potential for data to be shared with other researchers was one element in our research vignette; concerns about privacy may be activated in studies that involve sharing personal and/or genomic information with other investigators. Another possible explanation for the low participation intentions we found in the present study is because results from the research would not be provided. Retention in a clinical genetics research study was lower among African American women who did not receive a tangible benefit following study enrollment (e.g., genetic counseling for BRCA1 and BRCA2 mutations) [17]. Not receiving personal or research results may be a disincentive for participating in PMI among African Americans.

Although distrust has been shown to be a barrier to African American participation in clinical research [18,19] and is also associated with a reduced willingness to donate biospecimens to a biobank and participate in cancer genetics research [4], distrust did not have a significant independent association with the likelihood of participating in a government sponsored study that involved providing clinical data and biospecimens that would be used in the current study and may also be shared with other researchers to conduct future studies in the model that included overall participation barriers and facilitators. Altruistic values did not have a significant association with participation intentions in the logistic regression model with overall participation barriers and facilitators nor in the model that included specific participation barriers. However, in the model that included specific barriers related to lack of access to the study site, concern about who would be able to access personal health information, and not being provided with results from the study, distrust was associated with a significantly reduced likelihood of participating in the study described in the vignette. Conceptually, distrust has been described as a barrier to accepting enrollment [20] whereas having difficulty getting to the study site may reduce one’s opportunity to participate. Although the association was not statistically significant, respondents who reported that having difficulty getting to where the study was being conducted would make it unlikely that they would participate had a 50% lower likelihood of participating in study described in our vignette. Previous research has shown that African Americans who usually receive medical care from at a facility other than a doctor’s office (e.g., community center) were more likely to be willing to donate a biospecimens to cancer genetics research [5]. Our finding suggests that it may be important to include diverse types of health care settings in the PMI in order to ensure that racial minorities have a sufficient opportunity to participate in this research.

In both logistic regression models, men had an 80% increased likelihood of participating in the precision medicine study described in our vignette. African American men may be under-represented in clinical research [21], but there are some examples of successful recruitment of African American men in both genetic and non-genetic studies. Relative to women, African American men had similar rates of participating in a lifestyle intervention that was designed to increase motivation to change diet and physical activity [22]. Similarly, the African American Hereditary Prostate Cancer (AAHPC) [23] study enrolled 92 African American high risk families and complete clinical data were available on 154 African American men who had a personal history of prostate cancer [24]. Further, the Jackson Heart Study (JHS) enrolled 96% of the targeted sample of African American men and women and participants were diverse in terms of education, income, and employment characteristics [25]. The recruitment methods used in the JHS were developed based on the participation barriers and facilitators identified in research that was conducted prior to implementing study recruitment [25]. Common characteristics of previous studies that have been successful in recruiting African American men is that they examined participation barriers and facilitators prior to implementing recruitment activities, developed the study to demonstrate specific benefits to participation, and collaborated with diverse providers and organizations to implement recruitment [26–29]. Additional research is needed to develop a better understanding of the reasons why African American men would be willing to participate in PMI research and to translate this information into recruitment messages and strategies to increase the likelihood of enrollment. Although attitudes about participating in genetics research did not have a significant independent association with intentions to participate in a PMI study in the present report, these beliefs have been associated with intentions to donate biospecimens to a biobank [4]. In other research, we found that respondents who had greater participation facilitators relative to barriers were about twice as likely as those who had more participation barriers to participate in the genomic study described in our vignette.

A long-term goal for PMI is to develop a new model for conducting research that engages participants, shares data responsibly, and ensures privacy [30]. There is a growing body of research on barriers and facilitators to enrollment in cancer genetics research; our findings shed new light about how African Americans are likely to respond to an invitation to participate in PMI research. There was considerable variability in positive and negative expectations about participating in genetics research among respondents in the present study and the most important participation barriers included lack of access to the study site, concern about who would be able to access personal health information, and not being provided with results from the study. These issues can be addressed as part of efforts to develop precision medicine initiatives by including diverse types of health care settings as study sites and developing policies and procedures for data sharing and return of results that address the privacy concerns and preferences for research information among potential research participants. Given participation intentions did not vary among respondents based on their sociodemographic background or attitudes about participating in research, the way in which precision medicine studies are described to potential participants may be an important factor in participation decisions among African Americans. Additional research is needed to compare intentions to participate in genomic research that are described as having different objectives, benefits, and outcomes in order to determine the optimal ways to describe PMI studies to potential participants. These studies should also compare racial differences in responses to alternate research descriptions and determine if different factors are associated with the likelihood of participation among diverse groups. In addition to developing community and clinical partnerships to enhance PMI research, findings from the studies described above can be used to develop methods that enhance recruitment outcomes in racially and ethnically diverse group ethically. This form of research is critical. As PMI research becomes a national focus for improving prevention and treatment on an individual basis, a lack of representation of racial and ethnic minorities may limit the development of individualized strategies with populations that already experience a higher burden of chronic diseases and worse health care outcomes.

## Supporting Information

S1 Table Sample Characteristics. (XLSX)

S2 Table Attitudes about Participating in Genetics Research. (XLSX)

S3 Table Reasons for Participating in Cancer Genetics Research. (XLSX)

S4 Table Multivariate Logistic Regression Models of Participation. (XLSX)

## References

[1] Collins FS, Varmus H. A New Initiative on Precision Medicine. *N Engl J Med*. 2015;372:793–795. 10.1056/NEJMp1500523 25635347 PMC5101938

[2] Haga SB. Impact of limited population diversity of genome-wide association studies. *Genet Med*. 2010;12:81–84. 10.1097/GIM.0b013e3181ca2bbf 20057316

[3] McDonald JA, Barg FK, Weathers B, Guerra CE, Troxel AB, Domchek S, et al Understanding participation by African Americans in cancer genetics research. *J Natl Med Assoc*. 2012;104:324 23092046 10.1016/s0027-9684(15)30172-3 PMC3760677

[4] McDonald JA, Vadaparampil S, Bowen D, Magwood G, Obeid JS, Jefferson M, et al Intentions to donate to a biobank in a national sample of African Americans. *Public Health Genomics*. 2014;17:173–182. 10.1159/000360472 24942180 PMC5019027

[5] McDonald JA, Weathers B, Barg FK, Troxel AB, Shea JA, Bowen D, et al Donation intentions for cancer genetics research among African Americans. *Genet Test Molecular Biomarker*. 2012;16:252–258. 10.1089/gtmb.2011.0119 PMC3326272 22224593

[6] George S, Duran N, Norris K. A systematic review of barriers and facilitators to minority research participation among African Americans, Latinos, Asian Americans, and Pacific Islanders. *Am J Public Health*. 2014;104:e16–e31. 10.2105/AJPH.2013.301706 PMC3935672 24328648

[7] National Institutes of Health. The Precision Medicine Initiative Cohort Program—Building a Research Foundation for 21st Century Medicine. Precision Medicine Initiative (PMI) Working Group Report to the Advisory Committee to the Director, NIH. Available: https://www.nih.gov/precision-medicine-initiative-cohort-program/pmi-working-group

[8] Bussey-Jones J, Garrett J, Henderson G, Moloney M, Blumenthal C, Corbie-Smith G. The role of race and trust in tissue/blood donation for genetic research. *Genet Med*. 2010;12:116–121. 10.1097/GIM.0b013e3181cd6689 20098329 PMC3114600

[9] Bussey-Jones J, Henderson G, Garrett J, Moloney M, Blumenthal C, Corbie-Smith G. Asking the right questions: views on genetic variation research among black and white research participants. *J Gen Intern Med*. 2009;24:299–304. 10.1007/s11606-008-0883-7 19101773 PMC2642575

[10] Henderson G, Garrett J, Bussey-Jones J, Moloney ME, Blumenthal C, Corbie-Smith G. Great expectations: views of genetic research participants regarding current and future genetic studies. *Genet Med*. 2008;10:193–200. 10.1097/GIM.0b013e318164e4f5 18344709

[11] Smith TW. Altruism in contemporary America: A report from the National Altruism study. Chicago, IL: National Opinion Research Center; 2003.

[12] Pew Research Institute. Assessing the representativeness of public opinion surveys. Available: http://www.people-press.org/2012/05/15/assessing-the-representativeness-of-public-opinion-surveys/.

[13] Halbert CH, Kessler L, Stopfer JE, Domchek S, Wileyto EP. Low rates of acceptance of BRCA1 and BRCA2 test results among African American women at increased risk for hereditary breast-ovarian cancer. *Genet Med*. 2006;8:576–582. 16980814 10.1097/01.gim.0000237719.37908.54

[14] Kessler L, Collier A, Brewster K, Smith C, Weathers B, Wileyto EP, et al Attitudes about genetic testing and genetic testing intentions in African American women at increased risk for hereditary breast cancer. *Genet Med*. 2005;7:230–238. 15834240 10.1097/01.gim.0000159901.98315.fe

[15] McQuillan GM, Porter KS. Consent for future genetic research: the NHANES experience in 2007–2008. *IRB*. 2011;33:9–14. 21314035

[16] McQuillan GM, Porter KS, Agelli M, Kington R. Consent for genetic research in a general population: the NHANES experience. *Genet Med*. 2003;5:35–42. 12544474 10.1097/00125817-200301000-00006

[17] Halbert CH, Love D, Mayes T, Collier A, Weathers B, Kessler L, et al Retention of African American women in cancer genetics research. *Am J Med Genet*. 2008;146:166–173. 10.1002/ajmg.a.32067 18076114

[18] Corbie-Smith G, Thomas SB, St George DM. Distrust, race, and research. *Arch Intern Med*. 2002;162:2458–2463. 12437405 10.1001/archinte.162.21.2458

[19] Corbie-Smith G, Thomas SB, Williams MV, Moody-Ayers S. Attitudes and beliefs of African Americans toward participation in medical research. *J Gen Intern Med*. 1999;14:537–546. 10491242 10.1046/j.1525-1497.1999.07048.x PMC1496744

[20] Ford JG, Howerton MW, Lai GY, Gary TL, Bolen S, Gibbons MC, et al Barriers to recruiting underrepresented populations to cancer clinical trials: a systematic review. *Cancer*. 2008;112:228–242. 18008363 10.1002/cncr.23157

[21] Newton RL Jr., Griffith DM, Kearney WB, Bennett GG. A systematic review of weight loss, physical activity and dietary interventions involving African American men. *Obes Rev*. 2014;15:93–106. 10.1111/obr.12209 25196408

[22] Halbert CH, Bellamy S, Briggs V, Bowman M, Delmoor E, Johnson JC, et al Intervention completion rates among African Americans in a randomized effectiveness trial for diet and physical activity changes. *Cancer Epidemiol Biomarker Prev*. 2014;23:1306–1313. 10.1158/1055-9965.EPI-13-1064 PMC4445406 24755713

[23] Powell IJ, Carpten J, Dunston G, Kittles R, Bennett J, Hoke G, et al African-American heredity prostate cancer study: a model for genetic research. *J Natl Med Assoc*. 2001;93:120 12653398 PMC2593987

[24] Ahaghotu C, Baffoe-Bonnie A, Kittles R, Pettaway C, Powell I, Royal C, et al Clinical characteristics of African-American men with hereditary prostate cancer: the AAHPC Study. *Prostate Cancer Prostatic Dis*. 2004;7:165–169. 15175665 10.1038/sj.pcan.4500719

[25] Fuqua SR, Wyatt SB, Andrew ME, Sarpong DF, Henderson FR, Cunningham MF, et al Recruiting African-American research participation in the Jackson Heart Study: methods, response rates, and sample description. *Ethn Dis*. 2005;15:S6–18. 16317982

[26] Wyatt SB, Diekelmann N, Henderson F, Andrew ME, Billingsley G, Felder SH, et al A community-driven model of research participation: the Jackson Heart Study participant recruitment and retention study. *Ethn Dis*. 2003;13:438–455. 14632263

[27] Kennedy BM, Harsha DW, Bookman EB, Hill YR, Rankinen T, Rodarte RQ, et al Challenges to recruitment and retention of African Americans in the gene-environment trial of response to dietary interventions (GET READI) for heart health. *Health Educ Res*. 2011;26:923–936. 10.1093/her/cyr061 21865154 PMC3168336

[28] Parra-Medina D, D'Antonio A, Smith SM, Levin S, Kirkner G, Mayer-Davis E. Successful recruitment and retention strategies for a randomized weight management trial for people with diabetes living in rural, medically underserved counties of South Carolina: the POWER study. *J Am Diet Assoc*. 2004;104:70–75. 14702587 10.1016/j.jada.2003.10.014

[29] Yancey AK, Ortega AN, Kumanyika SK. Effective recruitment and retention of minority research participants. *Ann Rev Public Health*. 2006;27:1–28. 16533107 10.1146/annurev.publhealth.27.021405.102113

[30] National Institutes of Health. Precision Medicine Initiative. Available: http://www.nih.gov/precisionmedicine/.

